# A Review of Semen Analysis: Updates From the WHO Sixth Edition Manual and Advances in Male Fertility Assessment

**DOI:** 10.7759/cureus.63485

**Published:** 2024-06-29

**Authors:** Sunil Chawre, Mahalaqua Nazli Khatib, Alka Rawekar, Sanket Mahajan, Ritesh Jadhav, Akash More

**Affiliations:** 1 Physiology, Jawaharlal Nehru Medical College, Datta Meghe Institute of Higher Education and Research, Wardha, IND; 2 School of Epidemiology and Public Health, Jawaharlal Nehru Medical College, Datta Meghe Institute of Higher Education and Research, Wardha, IND; 3 Clinical Embryology, Datta Meghe Institute of Higher Education and Research, Wardha, IND

**Keywords:** dna fragmentation, semen parameters, who guidelines, male fertility, semen analysis

## Abstract

Semen analysis is essentially used to check the fertility of a man, especially when couples are having difficulties conceiving. Studies concerning male fertility, testicular factors, and seminal characteristics have been under investigation for the last few decades. In 1980, the World Health Organization (WHO) started reaching out to scientists in order to set standards for high-quality semen and develop a semen manual. From this point to the present, six editions of this manual have been produced, delineating the characteristics of semen and reporting protocols for semen analysis. Sperm morphology is analyzed as per WHO norms to measure the biological capacity of a male for reproduction. Both national and international manuals have been developed, with the latest, the sixth edition, produced in July 2021. This review paper conveys the current WHO publication's updates and identifies the clinical recommendations for proper evaluations. The publication considers the characteristics of semen in order to discuss the content of the previous editions of the WHO. It is also utilized to assess the method applied to determine the DNA presence of sperm fragmentation.

## Introduction and background

The inability to get pregnant after one year of unprotected intercourse is regarded as infertility. Infertility can be diagnosed and treated challengingly given that it has numerous potential causes, such as imbalances of hormones in males and females and irregularities in ovulation. The problem of infertility is acute; currently, one in every seven couples experiences difficulties in conception [1]. Semen analysis, commonly referred to as the sperm count test, is a test that is used to determine the quality of the sperm by checking the number, morphology, and kinetic functions of the sperm [2]. Abnormal sperm may be formed with defects in the head or tail region: a large-headed, malformed sperm or sperm with two tails or twisted tails. The size and shape of sperm relate to the morphological classification, which is significant in assessing male infertility [3]. For a normal test, about 50% of the sperm must be correctly motile after an hour of ejaculation since motility plays an essential role in fertilizing the egg [3].

The World Health Organization's (WHO) manual for examining semen is in its sixth edition. The current manual aims to give guidelines on methods that facilitate semen processing and give standardized instructions that help laboratories achieve uniformity in their results [4]. In elaborating on the WHO manual, it is important for stakeholders to ensure that the standards are clearly outlined and updated procedures instituted for the betterment of sexual and reproductive health [5]. In addition to the WHO manuals, numerous standard protocols for semen analysis have been developed by the International Organization for Standardization, and a number of the principles highlighted in the WHO guidelines are incorporated into these protocols. All these standards can help the laboratories that are aspiring for accreditation in semen examination. Accurate diagnosis can be time-consuming due to the complexity of the examination process [6]. The basic data set for the clinical examination of human semen derives from the WHO manuals and literature, where key parameters have been identified such as sperm count, morphology, and motility, which are essential in fertility testing [7].

Expert consensus indicates that the reliability, accuracy, and reproducibility of semen analysis results will only be guaranteed if set protocols are followed strictly. The following fertility-related issues can also influence the accuracy of the results: there is no universal method followed; the laboratory staff is not adequately trained; and the methods of testing may vary from region to region. All the above are fertility-related factors that influence the outcome of the test result [8]. Infertility checks may involve both the male and the female, although the most crucial role is usually taken by the female. Previous WHO guidelines used data collected from numerous laboratories; a large number of these laboratories demonstrated no reliability of their methods [9].

## Review

Review of semen assessment

The WHO Laboratory Manual for the Examination of Human Semen has been formulated to bring about consensus on laboratory methodologies for assessing semen since its first edition was launched in 1980. Since 1980, the WHO has promoted a standard procedure with the help of laboratory parameters and limit values, technical innovation inclusions, age, and representative samples that reflect the whole male population. To standardize the methods of semen analysis, the fifth edition issued in 2010 provided comprehensive, step-by-step instructions for both basic and optional tests in semen analysis. This also brought new standard guidelines on cryopreservation, which is crucial in fertility preservation and assisted reproductive technology (ART). It also incorporated facility specifications on testicular and epididymal sperm processing, hence enhancing uniformity among ART laboratories and clinical andrology. There was a suggestion to adhere to quality assurance measures that would ensure that the reports’ standards and the methods used to develop them were followed by the letter. Some key facets of the manual also encompassed illustrations involved in laboratory implementations and shared strategies for resolving common technical problems. Overall, the fifth edition significantly advanced the practice of male infertility from both clinical and research perspectives. It incorporated data from reference subsets in an effort to quantify male fertility. The reference ranges were from a multicenter study that classified semen parameters as fertile or infertile using the fifth centile of one-sided lower reference intervals. The study included 1959 males from eight different nations spread over four continents (Europe, the Americas, and Oceania).

However, the fifth edition faced criticism for its reference ranges of semen parameters as the primary measure for evaluating male fertility potential. Concerns were raised about the adequacy of these ranges to represent the general population due to the voluntary nature of cohort inclusion, geographic over- and under-representation, and biological and technical variations, including inconsistencies in quality assurance across laboratories. Critics, such as Björndahl et al., suggested using interpretation ranges instead of cut-off limits to assess fertility potential and raised concerns about lowering some reference ranges and the implications of omitting medical examinations in certain cases [10]. The sixth edition, released in July 2021, emphasized the importance of semen analysis as a tool for diagnosing fertility and infertility, assessing male reproductive health, guiding ART procedures, monitoring treatment responses, and evaluating the efficacy of male contraception. This edition aimed to optimize semen analysis procedures by providing detailed steps and methodological sequences for test execution. It introduced new sperm tests for assessing sperm DNA fragmentation (SDF) and seminal oxidative stress while discontinuing outdated tests like human cervical mucus. Additionally, the sixth edition addressed the demographic under-representation issues of the fifth edition by including data from fertile males whose partners had a time to pregnancy ≤12 months, collected between 2010 and 2020. The WHO manual of 2020 provides detailed guidelines for semen analysis and its nomenclature to assess male fertility. A summary of the key parameters and their reference values is shown in Table 1.

**Table 1:** Semen nomenclature according to WHO 2021 WHO: World Health Organization

Nomenclature	Definition
Azoospermia	Absence or complete lack of spermatozoa in the ejaculate
Oligozoospermia	Sperm count less than 15 million per ml
Teratozoospermia	having less than 4% of sperm with normal morphology
Asthenoteratozoospermia	having a count of total motile sperm less than 42%, or that of progressive motile sperm less than 30%
Oligoasthenoteratozoospermia (OAT)	less than 15 million sperm/mL, less than 42% total motility, and less than 4% of normal morphology
Oligoasthenozoospermia	Having less than 4% normal morphology of sperm and less than 15 million sperm per ml
Oligoteratozoospermia	Having less than 4% normal morphology and less than 15 million sperm per ml
Asthenoteratozoospermia	having less than 42% of all motile sperm and less than 4% of sperm with normal morphology
Normozoospermia	having semen parameters (≥15 million sperm/mL, ≥40% overall motility, and ≥4% normal morphology)
Severe OAT	Total no of sperm <5 million sperm/ml, with <4% morphology and <32% progressive motility
Severe oligoteratozoospermia	Total no of sperms <5% millions/ml

This new data included 3589 fertile males (1800 from the fifth edition and 1789 new subjects) from regions previously under-represented, such as Southern Europe, Asia, and Africa. However, Sub-Saharan Africa and South America are still under-represented in this edition. The sixth edition places a strong emphasis on stronger quality control procedures and improved standardization to ensure accuracy and similarity throughout labs. This covers thorough procedures for technician training, equipment calibration, and outside quality evaluation programs. A significant change in the sixth edition is the abandonment of reference values as definitive measures. It clarifies that the fifth centile values are only one method to interpret semen analysis results and are not sufficient alone to diagnose male infertility. The major differences between the fifth and sixth editions are outlined in Table 2.

**Table 2:** Comparative data between the fifth and sixth editions of WHO semen analysis procedures WHO: World Health Organization

Parameter	Fifth edition	Sixth edition
Sample collection	Standardized procedures	Updated standardized procedures
Initial examination	Basic macroscopy	Detailed macroscopy and investigation
Sperm motility	Basic assessment	Detailed motility and vitality check
Sperm count	Included	Included with more precision
Non-sperm cells count	Basic procedure	Advanced counting methods
Biochemical analysis	Limited	Comprehensive accessory sex organ function evaluation

Table 3 below compares the values between the fifth and sixth editions of the WHO semen analysis procedures.

**Table 3:** Comparison of WHO 2010 (fifth edition) and 2020 (sixth edition) parameters WHO: World Health Organization

Parameters	Fifth edition	Sixth edition
The volume of semen (mL)	1.4-1.7	1.3-1.5
Sperm motility percentage (%)	38-42	40-43
Non-motile sperm percentage (%)	21-22	19-20
Vitality percentage (%)	55-63	50-63
Motility for the case of nonmotility in the progression	Approximately one	1-1.1

Excerpts from the WHO manual

WHO has published a manual on human semen analysis and processing. Part one of the manual addresses semen analysis and the standard procedures for sample collection, followed by an initial macroscopic examination and further investigation [11]. Subsequent steps include assessing sperm motility, vitality, and count, as well as counting non-sperm cells. The manual also discusses optional biochemical procedures for evaluating accessory sex organ functions [12].

Semen analysis

Analysis Factors

Semen analysis is vital for identifying male infertility parameters. Accurate methods are available to assess sperm quality, including sperm count and morphology, though these factors alone are not highly predictive of pregnancy [13]. The analysis focuses on determining the fertilizing potential of spermatozoa. Various reproductive organ functions in females and certain technologies must be considered for predicting fertilization capacity and progeny behavior [14].

Comparison of Factors

The WHO's sixth edition manual recommends comprehensive semen analysis to diagnose fertility or infertility parameters [15]. It includes assessments of the male reproductive system and the effectiveness of male contraceptives. This edition provides a step-by-step procedure for semen analysis [16]. Table 2 compares parameters between the fifth and sixth editions of the WHO semen analysis procedures. The sixth edition of the manual introduces several new parameters and adjustments. One notable addition is the evaluation of semen odor, a complex parameter that necessitates laboratory safety precautions for handlers.

Parameters for analysis

Sperm Motility

The four-grade system that was previously employed in prior editions of the book has been replaced with two grades, A and B, for sperm motility in the sixth edition [17]. With this modification, assessments should be easier to understand and more accurate. Effectiveness in sperm counting is crucial for assessing sperm concentration, especially in samples with low concentrations (less than 2x10^6/ml) [18]. According to the sixth edition, an appropriate study of sperm vitality requires that the total motile sperm be less than 40%. High-quality semen samples are accurately categorized as normal or abnormal by following the manual's comprehensive, step-by-step procedure for evaluating sperm morphology [19-20].

Fertility

The sixth edition provides complete guidance regarding genetic assessment in relation to male factor infertility. This offers rich illustrations of the tests’ outcomes with less elaboration, as seen in the fifth edition, which failed to address the female partner’s age [21]. Prior research revealed that conception potential reaches the optimal rate at 29 years for females and thus included the female age as a component of fertility tests [22]. Also, the new manual has several new chapters and new sections on global methods such as reactive oxygen species (ROS) and a new examination section for ROS test methods. They are essential for an understanding of oxidative stress and its effects on the spermatogenic process and male fertility, as well as on the female genital organs [23-24].

The sixth edition also provides recommendations for analyses of SDF, applying methods such as the sperm chromatin dispersion test [25-26]. Single- or double-stranded DNA breaks are known as single- or double-stranded breaks (SDF), and they have been linked with a lower incidence of spontaneous abortions, inferior outcomes using ART, a lower pregnancy rate, diminished male fertility and ability, and low embryo quality [27].

Comparison with the earlier edition

Parameters

The fifth edition of the WHO manual focused on a population of 1,800 fertile males, establishing normal parameters with a fifth percentile value. This approach sparked controversy and objections regarding discrimination between fertile and infertile males [28]. The sixth edition addresses this by providing more comprehensive reference limits for both fertility and infertility [29]. The 2021 manual extends the interpretation of semen analysis results to include considerations for infertile men, a shift from the previous focus solely on fertile populations. It also offers guidelines for clinical decision-making in testing and treatment, which were lacking in earlier editions [30-31].

Importance of Semen Analysis and Artificial Intelligence in Semen Analysis

Semen analysis is fundamental for evaluating male fertility and spermatogenesis. Given its complexity, it should be conducted in a specialized andrology laboratory by well-trained technicians. Quality control is critical, both internally and externally, to ensure reliable test results. Evaluating sample variation is also essential before proceeding with the analysis [32-33]. Artificial intelligence and machine learning algorithms are being integrated into semen analysis, offering automated and highly accurate assessments of sperm motility, morphology, and concentration. These technologies reduce human error and increase diagnostic efficiency [34].

Treatment of Infertility

Over the last decade, significant developments have been made in infertility treatment, as reflected in the latest WHO manual on semen analysis. Key advancements include the use of computer-assisted sperm analysis and DNA fragmentation testing, which enhance the accuracy and consistency of semen quality assessments across different laboratories [35-36]. Compared to previous editions, the sixth edition of the WHO manual excludes less relevant chapters, such as those on cervical sperm interaction. It now mandates the storage of at least 10 or more semen samples for insemination procedures [37-38]. The cryopreservation of live spermatozoa is crucial, especially since even a single spermatozoon can be sufficient for fertilization. Proper storage is recommended before any sterilization procedures or therapies, such as radiotherapy or treatments involving alkylating agents, to prevent mutagenesis risks in spermatozoa [39-40].

Small amounts of leukocytes in semen can produce much higher levels of ROS than spermatozoa, potentially complicating sperm analysis due to chemiluminescence signals from sperm suspensions [41-42]. This highlights the importance of avoiding leukocyte contamination to prevent misleading results and issues with ejaculation [43]. Accessory gland secretions during ejaculation must be considered for their secretory function, requiring volume multiplication by the concentration in the accessory gland [44]. Abnormal results in male and female semen and mucus samples necessitate comprehensive testing of donor semen and cervical mucus [45-46]. No studies have explored sperm motility in viscous media. For mucus production, nitrogenization via ethinylestradiol administration to females for 6-10 days is suggested [47-48]. The European Association of Urology's 2021 guidelines recommend that both partners undergo fertility assessments, highlighting that male fertility evaluation should extend beyond semen analysis. Advanced technologies such as genetic evaluation, transrectal ultrasound, and retrograde ejaculation assessment are also recommended [49, 10].

## Conclusions

This review examines human semen analysis according to the instructions in the sixth edition of the WHO manual. It considers various parameters for assessing fertility, comparing them with those in the fifth edition. The sixth edition introduces advanced techniques for semen examination and improvements in sperm preparation and cryopreservation. This work discusses these advancements and their global impact on managing male fertility. The manual aims to support infertile couples by addressing potential male infertility issues.
